# Contactless Measurement of Sheet Resistance of Nanomaterial Using Waveguide Reflection Method

**DOI:** 10.3390/ma13225240

**Published:** 2020-11-19

**Authors:** Ming Ye, Raja Usman Tariq, Xiao-Long Zhao, Wei-Da Li, Yong-Ning He

**Affiliations:** 1 Faculty of Electronic and Information Engineering, Xi’an Jiaotong University, Xi’an 710049, China; raja_usman_tariq@stu.xjtu.edu.cn (R.U.T.); zhaoxiaolong@mail.xjtu.edu.cn (X.-L.Z.); boysid@stu.xjtu.edu.cn (W.-D.L.); yongning@mail.xjtu.edu.cn (Y.-N.H.); 2 State Key Laboratory of Millimeter Wave, Nanjing 210096, China

**Keywords:** microwave reflection, sheet resistance, noncontact, conductive glass

## Abstract

Conductive nanomaterials are widely studied and used. The four-point probe method has been widely used to measure nanomaterials’ sheet resistance, denoted as $R_{s}$. However, for materials sensitive to contamination or physical damage, contactless measurement is highly recommended if not required. Feasibility of $R_{s}$ evaluation using a one-port rectangular waveguide working on the microwave band in a contact-free mode is studied. Compared with existed waveguide methods, the proposed method has three advantages: first, by introducing an air gap between the waveguide flange and the sample surface, it is truly contactless; second, within the specified range of $R_{s}$, the substrate’s effect may be neglected; third, it does not require a matched load and/or metallization at the sample backside. Both theoretical derivation and simulation showed that the magnitude of the reflection coefficient $S_{11}$ decreased monotonously with increasing $R_{s}$. Through calibration, a quantitative correlation of $S_{11}$ and $R_{s}$ was established. Experimental results with various conductive glasses showed that, for $R_{s}$ in the range of ~10 to 400 Ohm/sq, the estimation error of sheet resistance was below ~20%. The potential effects of air gap size, sample size/location and measurement uncertainty of $S_{11}$ are discussed. The proposed method is particularly suitable for characterization of conductive glass or related nanomaterials with $R_{s}$ in the range of tens or hundreds of Ohm/sq.

## 1. Introduction

With the development of functional materials, applications for conductive nanomaterials were found in people’s daily lives, industry and scientific research. Specially, transparent conductive materials attract much attention due to their wide range of applications such as touch screens [1], displays [2], electromagnetic shielding [3], RF/microwave [4], glass heaters [5], solar cells [6], flexible electronics [7], OLED [8] and so on. In both science and engineering, sheet resistance $R_{s}$ is widely used to characterize a nanofilm’s electrical conductivity, and it is usually measured using the four-point probe method [9,10,11,12]. In this method, one major drawback is that good contact should be formed between the probes and the sample under test. This requirement may increase measurement cost and time, reduce efficiency and pollute or damage the nanomaterial under evaluation. Other contactless methods such as the eddy current method [13,14] and microwave method [15,16,17,18,19] were proposed as potential alternatives for sheet resistance evaluation.

Compared with the microwave method, the eddy current method is more widely accessible: one may find several commercially available instruments based on the principle of eddy current. It has been used in industry for decades. The microwave method usually works at a much higher frequency, e.g., from hundreds of MHz to hundreds of GHz or even THz (the eddy current method usually works in the KHz or low MHz band). The proposed method would be particularly suitable if one wants to study high-frequency conductivity.

There are two major categories for the sheet resistance measurement method based on microwave technology: the resonant method [18,19] and the nonresonant method [16,17]. In the resonant method, some kinds of resonant cavity can be used, such as a cylindrical resonator, dielectric resonator, or quasi-optical resonator, and a frequency-sweeping test is usually conducted to obtain the resonator’s quality factor. The publications of J. Krupka et al. are good references on this topic [20,21,22,23]. The sheet resistance of a nanofilm may also be measured by the nonresonant method, namely the transmission and/or reflection method. In this method, microwave transmission lines (such as waveguides or coaxial lines) or antennas (free space method) are used to detect the transmitted and/or reflected electromagnetic energy, which are quantitatively related to the sample’s sheet resistance.

Sheet resistance evaluation using waveguide has been proposed for decades, and it is still a research interest in which progress is being made. In the beginning, samples were needed to cut into specific dimensions to fill the waveguide cross-section [24,25]. Later, in the improved measurement setup, the samples were placed outside of the waveguide, but they needed good contact with the flange for easy parameter extraction [26,27,28,29]. Contactless measurement is possible by introducing an air gap between the sample and the flange. However, the backside of the sample should be metallic [30,31], or a matched load with a metal flange or dielectric flange is required [26].

In [16], a transmission method based on the dielectric waveguide was studied. However, it is more suitable for a millimeter wave frequency band such as that above 100 GHz. In [17], the authors correlate the transmission coefficient of the metallic waveguide to the metallic nanofilm’s sheet resistance. Although the method can work at a low frequency such as ~10 GHz, the major drawback is that it requires that the film should remain in good contact with waveguide flange. Otherwise, electromagnetic leakage induced by a small air gap may make the transmission coefficient much higher than in the case without leakage. Compared with the method described in [17], the proposed microwave reflection method in this work has the advantages of a sheet resistance measurement range (that is particularly suitable for transparent conductive materials), a simpler setup and a noncontact mode.

## 2. Materials and Methods

Several commercially available ITO conductive glasses were used as samples under test. All of the samples were of square shape with sides that were 100 mm in length (in fact, measurement accuracy of the proposed method may depend on the sample’s size relative to wavelength at the working frequency). Most of the samples had a thickness of 1.1 mm. To observe the possible effect of the substrate on the measurement results, samples with different thickness were also used. All of these samples were measured using a standard four-point probe (RTS-8, Guangzhou 4probes Technology, Inc., Guangzhou, China) and, for each sample, nine points in total were measured and averaged. The detailed information regarding the samples is summarized in Table 1. One can see from the four-point probe measurement results that most of the samples were quite uniform (sheet resistance varied ~1% across the whole surface; see the relative deviation column).

**Figure 1:**
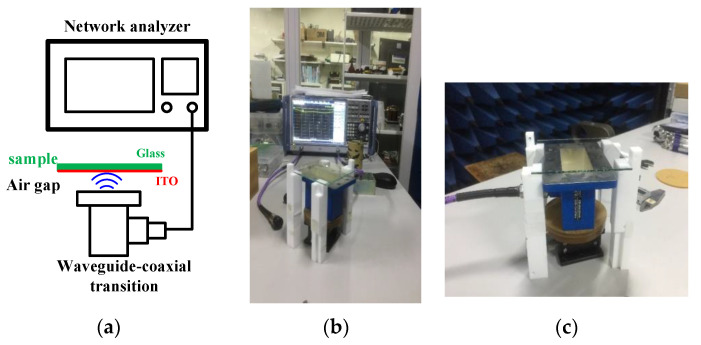
(**a**) Schematic of measurement setup; (**b**) photo of whole setup; (**c**) photo of waveguide sensor with sample.

A schematic view and photo of the measurement system used in this work are shown in Figure 1. A standard coaxial to waveguide transition (XB-WA284, Xibao Electronic Technology Co. Ltd., Beijing, China) was used as a transceiver. The waveguide was a standard WR284 waveguide with a standard flange. Microwave power was fed into the waveguide through the N type coaxial connector and it radiated onto the material under test. Depending on the sheet resistance of the nanomaterial, part of the microwave power will be reflected, as it was here. A network analyzer (ZND, Rohde & Schwarz GmbH & Co. KG, Munich, Germany) was used to measure the normalized reflection coefficient, namely S_11_ when transparent conductive glass was placed on the waveguide flange. Before sample measurement, the network analyzer was calibrated using a one-port reflected OSM routine to make the reference plane align with the coaxial connector of transition. Other parameters for VNA setup were: start frequency $f_{start}=2.2\;\mathrm{GHz}$, stop frequency $f_{stop}=4.5\;\mathrm{GHz}$; the input power was set to 0 dBm; the IF bandwidth for all the measurements was adjusted to 10 kHz; the averaging factor was 10. To restore the integrity of the signals reflected by the waveguide, the “Reduce Noise” option of the VNA was utilized.

## 3. Results and Discussion

**Figure 2:**
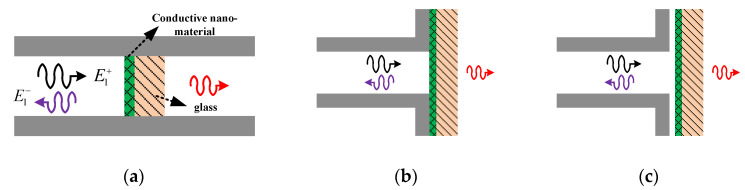
(**a**) Ideal model; (**b**) nonideal model for contact case; (**c**) nonideal model for contactless case.

Some theoretical formulation and simulation may be helpful for interpretation of measurement results. The case considered in the theoretical derivation is shown in Figure 2a and the other two cases shown in Figure 2b,c are considered in simulations. For the ideal case, the sample under test is embedded in a rectangular waveguide. Two methods are available for deriving the transmission/reflection coefficient for this case: the boundary condition method and network theory [25,27,28]. For the ideal case, starting from the boundary condition (one may refer to [17]), one can obtain the reflection coefficient as follows:

(1) $$R=\frac{\left[A_{1}\left(1-\beta_{1}/\beta_{2}\right)+A_{3}\left(1+\beta_{2}/\beta_{1}\right)\right]C_{1}+\left[A_{2}\left(1-\beta_{2}/\beta_{1}\right)+A_{4}\left(1+\beta_{2}/\beta_{1}\right)\right]C_{2}}{\left[A_{1}\left(1+\beta_{2}/\beta_{1}\right)+A_{3}\left(1-\beta_{2}/\beta_{1}\right)\right]C_{1}+\left[A_{2}\left(1+\beta_{2}/\beta_{1}\right)+A_{4}\left(1-\beta_{2}/\beta_{1}\right)\right]C_{2}},$$

Here, $A_{1}=\left(1+\beta_{3}/\beta_{2}\right)exp\left[j\left(\beta_{2}-\beta_{3}\right)t_{2}\right],$ $A_{2}=\left(1-\beta_{3}/\beta_{2}\right)exp\left[j\left(\beta_{2}+\beta_{3}\right)t_{2}\right],$ $A_{3}=\left(1-\beta_{3}/\beta_{2}\right)exp\left[-j\left(\beta_{2}+\beta_{3}\right)t_{2}\right],$ $A_{4}=\left(1+\beta_{3}/\beta_{2}\right)exp\left[-j\left(\beta_{2}-\beta_{3}\right)t_{2}\right],$ $C_{1}=\left(1+\beta_{1}/\beta_{3}\right)exp\left[j\left(\beta_{3}-\beta_{1}\right)\left(t_{2}+t_{3}\right)\right],$ $C_{2}=\left(1-\beta_{1}/\beta_{3}\right)exp\left[-j\left(\beta_{3}+\beta_{1}\right)\left(t_{2}+t_{3}\right)\right],$ $\beta_{1}=\sqrt{-{\left(\frac{\pi }{a}\right)}^{2}+\omega^{2}\mu_{0}\varepsilon_{0}},$ $\beta_{2}=\sqrt{-{\left(\frac{\pi }{a}\right)}^{2}+\omega^{2}\mu_{0}\varepsilon_{0}-j\omega \mu_{0}\sigma },$ $\beta_{3}=\sqrt{-{\left(\frac{\pi }{a}\right)}^{2}+\omega^{2}\mu_{0}\varepsilon_{0}\varepsilon_{r,sub}\left(1-j\tan\delta \right)},$ a is the width of the waveguide, ω is the angular frequency, $\mu_{0}$ and $\varepsilon_{0}$ are the vacuum permeability and permittivity, respectively, σ is the conductivity of the conductive nanofilm and $\varepsilon_{r,sub}$ and tanδ are the relative dielectric constant and the loss tangent of the substrate, respectively. If the substrate can be neglected, then Equation (1) can be simplified to

(2) $$R=\frac{A_{1}\left(1-\beta_{1}/\beta_{2}\right)+A_{3}\left(1+\beta_{2}/\beta_{1}\right)}{A_{1}\left(1+\beta_{2}/\beta_{1}\right)+A_{3}\left(1-\beta_{2}/\beta_{1}\right)}.$$

Furthermore, assuming that $\beta_{1}\ll \beta_{2}$ and $\beta_{2}t_{2}\ll 1$, then

(3) $$R=\frac{\omega \mu_{0}}{2\beta_{1}R_{s}+\omega \mu_{0}}.$$

Here, $R_{s}=1/\left(\sigma t\right)$ is the sheet resistance of the nanomaterial which is determined by the conductivity σ and thickness t. Thus, it is expected that one can estimate sheet resistance by measuring the reflection coefficient. Equation (2) can be written as

(4) $$S_{11,dB}=20\log\left(\frac{\omega \mu_{0}}{2\beta_{1}R_{s}+\omega \mu_{0}}\right).$$

According to [17] (in this publication, it is claimed that the transmission method is suitable for samples with sheet resistance ranging from ~50 to 500 mOhm/sq), the simplified transmission coefficient can be represented as:

(5) $$S_{21,dB}=20\log\left|\frac{2\gamma_{1}}{\omega \mu_{0}}R_{s}\right|.$$

**Figure 3:**
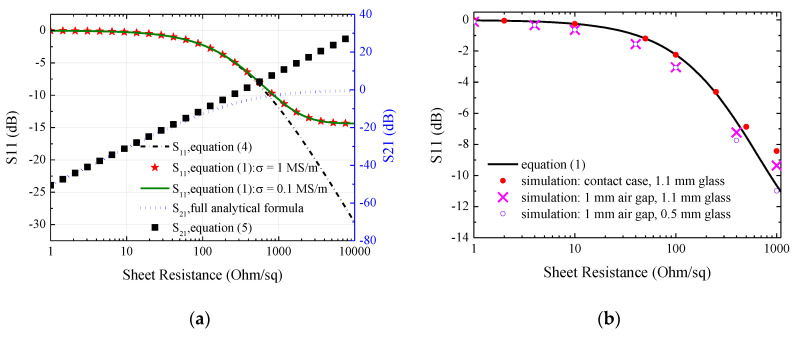
(**a**) Analytical calculations for ideal case; (**b**) simulations for nonideal cases. Default conductivity for nanofilm is 1 MS/m, glass thickness is 1.1 mm, and working frequency is 2.5 GHz. The curve, denoted as “S_11_, full analytical formula”, can be obtained using Equation (12) from [17].

The calculation results of the ideal case shown in Figure 2a are presented in Figure 3a. The inset figure shows the reflection coefficient for the sheet resistance below 10 Ohm/sq. It can be seen for both the transmission and reflection coefficients that the simplified equation agrees with the rigorous formula when the sheet resistance is below ~100 Ohm/sq and ~500 Ohm/sq, respectively. Considering the measurement accuracy of S_11_, one may expect that the reflection method is most suitable for samples with sheet resistance in the range of ~10 to 1000 Ohm/sq. Furthermore, if one wants to exclude the possible effect of the substrate, the measurement range may be confined in the range of ~10 to 400 Ohm/sq. The curve denoted as “0.1 MS/m” agrees well with the curve denoted as “Equation (1)” (the used conductivity of nanofilm is 1 MS/m), indicating that it is indeed the sheet resistance determining S_11_. It can then be expected that the proposed measurement method should be feasible for a variety of conductive thin film materials but not limited to the ITO samples mentioned above. It can be seen from Figure 3b that: 1. for all of the cases considered, the sheet resistance may be extracted from S_11_ due to their monotonous correlation; 2. there is some difference between the case “sample embedded in waveguide” (denoted as “Equation (1)”) and the case “sample out of waveguide but contact with flange” (denoted as “simulation: contact case, 1.1 mm glass”) only when the sheet resistance is larger than ~200 Ohm/sq; 3. when the sheet resistance is larger than ~100 Ohm/sq, the substrate’s thickness has an effect on S_11_. Thus, if one would like to neglect the substrate’s effect and S_11_ can be measured accurately, it can be expected that the proposed method may work well for nanomaterials with sheet resistance in the range of ~1–1000 Ohm. However, as described later, due to uncertainties in S_11_ measurement, the size of the air gap and other factors, experimental results in this work demonstrate a narrower measurement range of sheet resistance.

**Figure 4:**
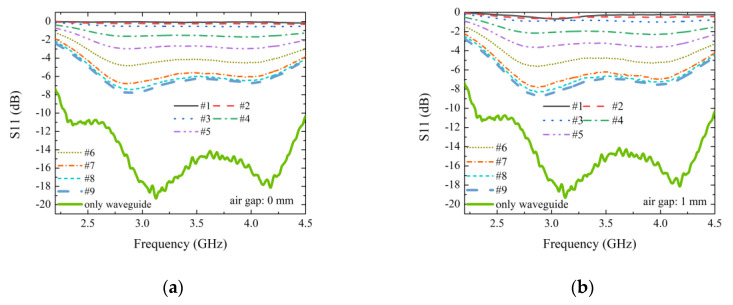
Measured normalized reflection coefficient (S_11_) for all of the samples with glass thickness 1.1 mm: (**a**) without air gap; (**b**) with 1 mm air gap.

The measurement results of the samples with thickness of 1.1 mm are shown in Figure 4; the contact case is shown in Figure 4a and the noncontact case (an air gap of 1 mm was introduced between waveguide flange and sample) is shown in Figure 4b. It should be noted that, for the contact case, we just placed the sample on the flange without any additional applied load. The measured S_11_ when no sample is presented is also shown. This curve may be seen as the noise floor, and it can be seen in the measured frequency band that the S_11_ is almost below −10 dB. This is because the WR284 waveguide has a characteristic impedance $Z_{0}$ close to air. Namely, according to the theory of the rectangular waveguide, $Z_{0}\;=\;\pi bZ_{0}\omega \mu_{0}/(2\alpha \beta )$, here, a and b are the waveguide width and height, respectively, ω is the angular frequency, $\mu_{0}$ is the vacuum permeability and β is the propagation constant. For example, at 2.5 GHz, the waveguide used here has its characteristic impedance of about 500 Ohm, which is close to the air impedance, 377 Ohm. For both the contact case and the noncontact case, the reflection coefficient decreases with increasing resistance. Namely, a higher sheet resistance results in lower reflection. This can be attributed to higher transmission and absorption of high resistance samples. One may also notice that dependence of S_11_ on sheet resistance is influenced by the working frequency. However, a consistent correlation between S_11_ and sheet resistance can be established at almost any frequency point in the measured frequency band.

**Figure 5:**
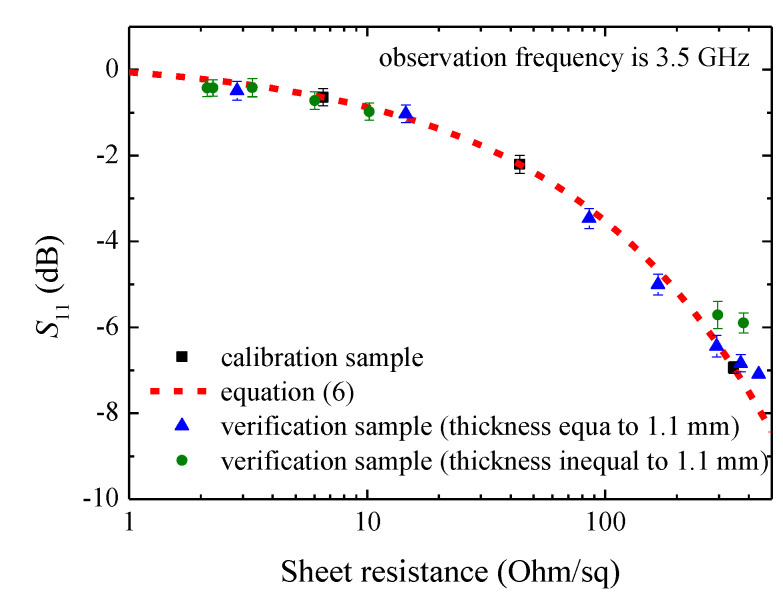
Measured dependence of S_11_ on sheet resistance at 3.5 GHz for all of the samples. The fitting curve calculated using Equation (6) is also shown for comparison.

Due to electromagnetic field leakage between the waveguide flange and the sample as well as possible setup assembly error, the measured S_11_ of our experimental setup was slightly different from that of the above theoretical results. Compared with simulations in which a standard rectangular waveguide transmission line was used, our experimental setup was based on a coaxial-to-waveguide transition. Thus, comparing them directly was not rigorous. However, if one calibrated the VNA with Through-Reflect-Line (TRL) techniques, then the reference plane could be positioned at the waveguide flange. In this case, one could compare the simulation with the measurement. From the measurement point of view, if a quantitative correlation could be established between the sheet resistance and S_11_, then it was possible to estimate the sheet resistance from the measured S_11_. Through mathematic fitting, we found that the power function $S_{11,dB}=c_{01}R_{s}^{c_{02}}+c_{03}$ was suitable for this purpose. Since three unknowns were presented in the power function, we selected three samples of thickness 1.1 mm as calibration sample as shown in Figure 5, and the obtained fitting coefficients were:

(6) $$S_{11,dB}=-0.3615R_{s}^{0.5127}+0.3038.$$

It should be noted that Equation (6) was obtained based on S_11_ observed at 3.5 GHz (we also tried with other frequencies and similar results were obtained). Using Equation (6), we predicted other samples’ (including samples with thickness of 1.1 mm or other thickness) sheet resistance values and compared them with the four-point probe measurements, as shown in the last two columns of Table 1. It can be seen that, for all of the samples with thickness of 1.1 mm, except for samples #1 and #10, the prediction error was below 15%. The relatively large error for sample #1 may be attributed to the measurement uncertainty of S_11_ as described later. For sample #10, it was on top of the fitting curve and caused the whole measurement curve to exhibit a saturate tendency. This agrees with the results shown in Figure 3. Namely, when sheet resistance is large, the substrate’s effect will become obvious, or it can be claimed that S_11_ is determined by the substrate’s properties such as permittivity and thickness. Thus, it may be concluded that the proposed contactless method is suitable for samples with sheet resistance in between ~10 to ~400 Ohm/sq. For samples with thickness other than 1.1 mm, we also observed that the total estimation error can be below 20% if sheet resistance is between ~10 to ~400 Ohm/sq (see, for example, samples #14 and #15). Sample #16 had a relatively large estimation error which may be attributed to its large sheet resistance. For industry application, since nanomaterials are produced in large volume with the same substrate, mathematic fitting can be applied for each special substrate to improve estimation accuracy.

**Figure 6:**
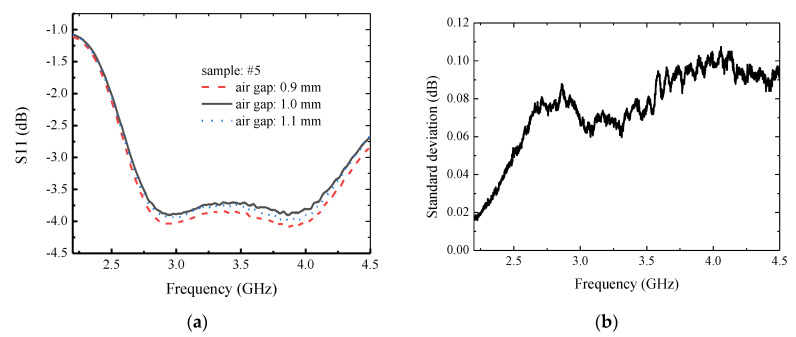
Measured effect of air gap: (**a**) S_11_ vs. frequency; (**b**) standard deviation of S_11_.

Regarding uncertainty analysis, several potential factors were considered. First, the air gap size was controlled manually in our experiment. The error of the air gap size could have been ~100 microns. To observe the potential effect of air gap on measured S_11_, we conducted a group of measurements with a tuned air gap size. S_11_ measurement results are shown in Figure 6a and we also used the three curves in Figure 6a to calculate the standard deviation of the magnitude of S_11_ as shown in Figure 6b. It can be seen that when the air gap size change was +/− 0.1 mm, the change of S_11_ was almost below 0.1 dB. This may have had some effect on the estimation result of the sheet resistance. In fact, the measurement accuracy of S_11_ is claimed to be 0.1 dB by the network analyzer supplier. Thus, the observed standard deviation may have also been partly caused by the analyzer itself. Anyway, with controlled assembly accuracy, one may construct a setup with higher precision to minimize measurement uncertainty induced by air gap size error.

**Figure 7:**
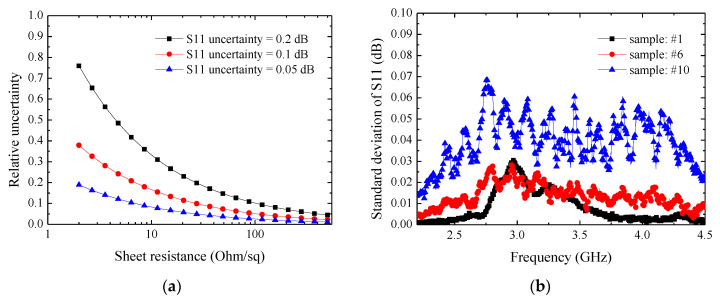
(**a**) Effect of the measurement uncertainty of S_11_ on sheet resistance evaluation; (**b**) effect of the horizontal position of samples on measured S_11_.

Using Equation (6), we analyzed the effect of measurement uncertainty of S_11_ on the sheet resistance prediction accuracy as shown in Figure 7a. It can be seen that: 1. measurement accuracy increased with decreasing S_11_ uncertainty; 2. measurement accuracy was better for higher resistance samples. These observations are helpful for understanding the measurement results of samples with sheet resistance below ~10 Ohm/sq. In Figure 7b, we present the measured effect of the horizontal place position of the sample on S_11_. We moved the sample along the *x*- and *y*-axes with 1 mm steps and 3 mm total displacement. With these nine measurements, we calculated the standard deviation. It can be seen that the change of S_11_ was below 0.1 dB. Thus, the result indicates that the proposed method is, to some degree, insensitive to placement locations. This shall be helpful for real application. It should be noted that the proposed method has a minimum requirement on sample size; this will be studied in near future. To summarize, the above uncertainty analysis may indicate that the major contribution of sheet resistance estimation error is coming from S_11_ measurement.

## 4. Conclusions

A noncontact sheet resistance measurement method based on microwave reflection was proposed and verified. Both theoretical derivations and simulations showed that the sheet resistance correlates well with the magnitude of reflection coefficient. Demonstrations with an open-ended waveguide also showed that the proposed method is suitable for contactless sheet resistance evaluation in the range of ~10 to 400 Ohm/sq; this makes the proposed method especially suitable for ITO samples. This will be particularly suitable for industry’s online evaluation or in situ monitoring of the thin film deposition process. In future, the effect of the substrate and the sample’s size will be studied systematically. Possible directions for performance improvement include use of a precise setup and a high-performance network analyzer or optimal analyzer setup parameters with higher measurement accuracy (e.g., use TRL calibration). Experimental verifications with other materials and waveguides working at higher frequency bands will be conducted in near future. The potential effects of the environmental conditions (such as humidity, air pressure and temperature) and the effect of the surface roughness of the thin film on measurements will also be included in future work.

## Figures and Tables

**Table 1:** Sheet resistance ($R_{s}$) of ITO samples measured using the four-point probe method and the microwave method.

Sample #	Thickness of Glass (mm)	$\mathrm{Averaged}\;\mathrm{Sheet}\;\mathrm{Resistance}\;R_{s}\;(\mathrm{Ohm}/\mathrm{sq}){\;}^{1}$	$\mathrm{Standard}\;\mathrm{Deviation}\;\mathrm{of}\;R_{s}\;(\mathrm{Ohm}/\mathrm{sq})$	Relative Deviation ^2^ (%)	$\mathrm{Sheet}\;\mathrm{Resistance}\;R_{s}\;(\mathrm{Ohm}/\mathrm{sq}){\;}^{3}$	Relative Error ^4^ (%)
1	1.1	2.841	0.029	1.02	4.593	61.7
2	1.1	6.531	0.041	0.62	-	-
3	1.1	14.53	0.109	0.75	12.70	−12.6
4	1.1	43.70	0.527	1.21	-	-
5	1.1	85.56	0.688	0.80	96.63	12.9
6	1.1	166.3	1.528	0.92	188.7	13.5
7	1.1	276.0	4.123	1.49	336.2	−9.16
8	1.1	293.7	2.500	0.85	300.7	2.40
9	1.1	346.0	6.500	1.88	-	-
10	1.1	441.4	7.986	1.81	359.8	−18.5
11	0.5	3.291	0.064	1.94	3.835	16.5
12	1.8	2.250	0.014	0.63	3.905	73.6
13	1.8	6.019	0.028	0.46	7.596	26.2
14	1.8	10.19	0.142	1.39	11.81	15.9
15	1.8	296.4	18.07	6.10	240.5	−18.9
16	1.8	380.8	8.105	2.13	255.4	−32.9
17	2.0	2.129	0.026	1.23	3.939	85.0

^1^ Four-point probe test. ^2^ Relative deviation is equal to standard deviation divided by average value. ^3^ Microwave test. ^4^ Relative error is obtained by comparing the microwave test with the four-point probe method.
